# Calibration of arterial spin labeling data—potential pitfalls in post‐processing

**DOI:** 10.1002/mrm.28000

**Published:** 2019-10-12

**Authors:** Joana Pinto, Michael A. Chappell, Thomas W. Okell, Melvin Mezue, Andrew R. Segerdahl, Irene Tracey, Pedro Vilela, Patrícia Figueiredo

**Affiliations:** ^1^ Institute for Systems and Robotics and Department of Bioengineering Instituto Superior Técnico Universidade de Lisboa Lisbon Portugal; ^2^ Wellcome Centre for Integrative Neuroimaging FMRIB Nuffield Department of Clinical Neurosciences University of Oxford Oxford United Kingdom; ^3^ Institute of Biomedical Engineering Department of Engineering Science University of Oxford Oxford United Kingdom; ^4^ Nuffield Division of Anaesthetics Nuffield Department of Clinical Neuroscience University of Oxford Oxford United Kingdom; ^5^ Imaging Department Hospital da Luz Lisbon Portugal

**Keywords:** ASL, calibration, cerebral blood flow, kinetic modeling, MRI

## Abstract

**Purpose:**

To assess the impact of the different post‐processing options in the calibration of arterial spin labeling (ASL) data on perfusion quantification and its reproducibility.

**Theory and Methods:**

Absolute quantification of perfusion measurements is one of the promises of ASL techniques. However, it is highly dependent on a calibration procedure that involves a complex processing pipeline for which no standardized procedure has been fully established. In this work, we systematically compare the main ASL calibration methods as well as various post‐processing calibration options, using 2 data sets acquired with the most common sequences, pulsed ASL and pseudo‐continuous ASL.

**Results:**

Significant and sometimes large discrepancies in ASL perfusion quantification were obtained when using different post‐processing calibration options. Nevertheless, when using a set of theoretically based and carefully chosen options, only small differences were observed for both reference tissue and voxelwise methods. The voxelwise and white matter reference tissue methods were less sensitive to post‐processing options than the cerebrospinal fluid reference tissue method. However, white matter reference tissue calibration also produced poorer reproducibility results. Moreover, it may also not be an appropriate reference in case of white matter pathology.

**Conclusion:**

Poor post‐processing calibration options can lead to large errors in perfusion quantification, and a complete description of the calibration procedure should therefore be reported in ASL studies. Overall, our results further support the voxelwise calibration method proposed by the ASL white paper, particularly given the advantage of being relatively simple to implement and intrinsically correcting for the coil sensitivity profile.

## 1 INTRODUCTION

Arterial spin labeling (ASL) is a non‐invasive MRI technique that provides quantitative images of tissue perfusion, by using magnetically labeled blood water protons as an endogenous blood flow tracer.^1^, ^2^, ^3^ ASL is acknowledged to have great potential as a completely non‐invasive quantitative perfusion imaging technique, but its implementation has been challenging because of the intrinsically low SNR. This has motivated the development of a multitude of signal acquisition and processing strategies that aim to overcome this limitation as well as the publication of a ASL implementation consensus paper.^4^ Although pseudo‐continuous ASL (pCASL) is the recommended labeling strategy, pulsed ASL (PASL) is still a commonly used technique.^5^, ^6^, ^7^, ^8^, ^9^

In principle, cerebral blood flow (CBF) can be quantified based on a single time delay measurement—the post‐labeling delay (PLD) in pCASL and the inversion time (TI) in PASL—provided that it is long enough relative to the transit time of the label bolus between the arteries and the capillaries, the so‐called arterial transit time (ATT). However, if this condition cannot be assumed, which is the case in pathologies presenting delayed arterial transit times, multiple‐PLD/TI measurements are necessary.^10^, ^11^, ^12^ Furthermore, if an appropriate kinetic model is fitted to the data, the multiple‐PLD/TI strategy allows the assessment of not only CBF but also ATT^13^ and potentially arterial blood volume (aBV),^14^, ^15^ which may be parameters of interest in their own right. In any case, to obtain CBF measures in absolute units, it is necessary that the relative CBF (*CBF_rel_*) images should be normalized by the equilibrium magnetization of arterial blood (*M*_0_ *_a_*), which is usually derived from the equilibrium magnetization measured in tissue (*M*_0_ *_t_*). Critically, it has been demonstrated through theoretical analysis that the 2 factors that CBF quantification using ASL is most sensitive to are *M*_0_ *_a_* estimation and labeling efficiency.^16^

The current recommendation for ASL calibration involves the acquisition of a separate proton‐density weighted image followed by the extraction of *M*_0_ *_t_* by extrapolation as a function of the repetition time (TR). This is subsequently converted to an *M*_0_ *_a_* image by first applying spatial smoothing and then dividing the image by the brain average brain–blood water partition coefficient (λ).^4^ Nevertheless, several other strategies might be used depending on the acquisition scheme. If no background suppression is used (e.g., PICORE),^17^ it is also possible to obtain *M*_0_ *_t_* directly from the ASL data by averaging the control images at a particular PLD/TI. In the specific case of multiple‐TI PASL, it is possible to obtain *M*_0_ *_t_* by fitting a saturation‐recovery curve to the multiple‐TI control images. Additionally to the selection of a calibration image, it is necessary to decide whether to compute a voxelwise *M*_0_ *_a_* value, or a single average value across a homogeneous reference region, usually gray matter (GM), white matter (WM), or cerebrospinal fluid (CSF).^18^, ^19^ For the practical implementation of the calibration pipeline, a number of subtler choices must also be made, however, these are rarely discussed or even reported in ASL studies. Although some calibration method comparisons have been reported,^16^, ^18^, ^19^, ^20^ the impact and potential pitfalls of the corresponding processing options have yet to be investigated.

Here, we systematically compare the impact of using different calibration post‐processing pipelines on the quantification of perfusion and its reproducibility, using both PASL and pCASL acquisitions.

## 2 THEORY

Calibration is required to obtain CBF (mL/100 g/min) in absolute units from ASL measurements, through normalization by *M*_0_ *_a_* (and α, the labeling/inversion efficiency)

(1) $$CBF=\frac{CBF_{\mathrm{rel}}}{\alpha M_{0a}}\times 6000.$$

Calibration therefore entails estimation of *M*_0_ *_a_* and it is achieved in 2 main steps: (1) generation of *M*_0_ *_t_* map, and (2) derivation of *M*_0_ *_a_* from *M*_0_ *_t_* map.

### 2.1 Generation of *M*_0_ *_t_* map

The method used for generating the *M*_0_ *_t_* map depends on the acquisition scheme and options chosen. There are 3 main strategies: long TR calibration scan (*LongTR*), ASL control averaging (*CtrAvg*), and control saturation recovery (*SatRec*).

#### 2.1.1 Long TR calibration scan (*LongTR*)

The *LongTR* approach is based on a separately acquired long TR scan, which approximates the equilibrium magnetization of tissue in each voxel i, *M*_0_ *_t_* (i). This map should be corrected for the amount of *T*_1_ relaxation at the TR at each voxel, according to

(2) $$M_{0t,corr}\left(i\right)=\frac{M_{0t}\left(i\right)}{1-e^{-\frac{TR_{\mathrm{long}}}{T_{1,t}}}},$$

where *T*_1,_ *_t_* corresponds to the tissue‐specific *T*_1_ in each voxel i and *TR_long_* is the corresponding TR of the *M*_0_ *_t_* scan. This correction depends on a separate tissue segmentation procedure. Using a long TR minimizes the impact of the *T*_1_ correction, which may become negligible for sufficiently long TR values.

#### 2.1.2 Averaging of control images (*CtrAvg*)

If no background suppression is applied, it is also possible to estimate *M*_0_ *_t_* based on the control images. This can be carried out by averaging control images at a fixed TI (*M_ctrl_ (i)*), which should then be corrected for the amount of *T*_1_ relaxation during TI, at each voxel, to yield a corrected map of the tissue equilibrium magnetization

(3) $$M_{0t,corr}\left(i\right)=\frac{M_{\mathrm{ctrl}}\left(i\right)}{1-Ae^{-\frac{\mathrm{TI}}{T_{1,t}}}}.$$

Furthermore, in this approach, the inversion–saturation efficiency (*A*) is also a variable. A value of 0.90 was chosen as it was the approximate average value of *A* estimated in *SatRec*.

For the generation of the *M*_0_ *_t_* map, one can argue that this method is less appropriate, mainly because the value of TI is not chosen for this purpose and therefore it is not sufficiently long to minimize the dependency on the values of *T*_1_ and *A*.

#### 2.1.3 Saturation recovery of control images (*SatRec*)

If no background suppression is applied, it is possible to estimate *M*_0_ *_t_* by fitting a saturation‐recovery curve to the series of control images, *M_ctrl_* (TI), if multiple TIs are sampled and the acquisition sequence includes presaturation (which is the case of Q2TIPS PASL).^17^ This strategy allows estimation of *M*_0_ *_t_* as well as *T*_1_ *_t_* and saturation efficiency (*A*) (“asl_calib”, https://fsl.fmrib.ox.ac.uk/fsl/fslwiki/BASIL/).

### 2.2 Derivation of *M*_0_ *_a_* from *M*_0_ *_t_* map

Once an *M*_0_ *_t_* map is generated, the next step is the derive *M*_0_ *_a_*. Two types of methods are commonly used for that purpose: the reference tissue (RT) and the voxelwise (Voxel).

#### 2.2.1 Reference tissue methods (RT‐CSF, RT‐WM, RT‐GM)

The reference tissue (RT) approach consists of computing a single *M*_0_ *_a_* value based on a homogeneous tissue region (GM, WM, or CSF). The first step is to correct the *M*_0_ *_t_* (as well as CBF) maps for the RF head coil sensitivity profile. In case the coil sensitivity profile is not available, an approximate correction may be carried out by normalizing the *M*_0_ *_t_* (and CBF) maps using a bias image estimated directly from the data using appropriate post‐processing tools (e.g., FAST).^21^

The next step is to define an appropriate RT mask across which an average *M*_0_ *_t_* value is computed, <*M*_0_ *_t_*>*_rt_*. This usually involves tissue segmentation based on a structural image (*T*_1_‐weighted image or *T*_1_ *_t_* map in case this has been estimated) followed by registration of the RT mask with the *M*_0_ *_t_* map. Finally, the *M*_0_ *_a_* value is obtained by normalizing <*M*_0_ *_t_*>*_rt_* with the respective λ, while correcting for the difference in $T_{2}^{*}$ relaxation between the reference tissue and arterial blood (associated with the *M*_0_ *_t_* measurement), yielding

(4) $$M_{0a}=\frac{<M_{0t}{>}_{\mathrm{rt}}e^{TE\left(\frac{1}{T_{2,rt}^{*}}-\frac{1}{T_{2,a}^{*}}\right)}}{\lambda_{\mathrm{rt}}},$$

where $T_{2,a}^{*}$ is the transverse relaxation time of arterial blood, $T_{2,\mathrm{rt}}^{*}$ is the transverse relaxation time of the reference tissue, and λ_rt_ is blood–brain water partition coefficient of the corresponding reference tissue.

#### 2.2.2 Voxelwise method (Voxel)

In this method, an *M*_0_ *_a_* map is obtained through direct extrapolation from the *M*_0_ *_t_* value in each voxel, by normalizing with the respective tissue λ, while correcting for the difference in $T_{2}^{*}$ relaxation between the respective tissue and arterial blood, according to

(5) $$M_{0a}\left(i\right)=\frac{M_{0t}\left(i\right)e^{TE\left(\frac{1}{T_{2,t}^{*}}-\frac{1}{T_{2,a}^{*}}\right)}}{\lambda_{t}},$$

where $T_{2,t}^{*}$ and λ_t_ are the transverse relaxation time and the blood–brain water partition coefficient, respectively, of the tissue in each voxel. Regarding λ_t_, in principle, a different λ value exists in each voxel because λ differs as a function of tissue type and hence the voxel’s partial volume estimates (PVEs). For that reason, λ_t_ should be a PVE‐weighted average of λ. PVEs are usually obtained through tissue segmentation using appropriate tools (e.g., FAST).^21^ The *M*_0_ *_a_* map is then smoothed, to minimize noise contributions.

### 2.3 Simplifications

A number of assumptions and approximations to the theoretically ideal calibration just described are usually undertaken to overcome additional complexities in both data acquisition and processing. These are described here for each of the main calibration methods, and the respective post‐processing options that are tested in this work are indicated, relative to the default options derived from the theoretical considerations above. All options are summarized in Table 1.

**Table 1:** Summary of calibration methods and post‐processing options tested

Method	Options		
**Generation of *M*_0_*_t_* map**			
PASL‐*SatRec*	presaturation efficiency	*A* estimated (default)	
		*A* = 100%	
PASL‐*CtrAvg*	TI value	long TI ~2400 ms (default)	
		short TI ~800 ms	
	*T*_1_ correction	yes (default)	
		no	
	presaturation efficiency	*A* = 90% (default)	
		*A* = 100%	
pCASL‐*LongTR*	*T*_1_ correction	yes (default)	
		no	
**Derivation of *M*_0_*_a_* from *M*_0_*_t_* map**			
Reference tissue CSF	RT mask	restrictive (default)	restrictive threshold (PVE_CSF_ ≥0.9) intersected with MNI atlas ventricles mask
		intermediate	less restrictive threshold (PVE_CSF_ ≥0.6) intersected with MNI atlas ventricles mask
		extensive	less restrictive threshold (PVE_CSF_ ≥0.6)
	bias correction	yes (default)	
		no	
Reference tissue WM	RT mask	restrictive (default)	restrictive threshold (PVE_WM_ ≥0.9)
		extensive	less restrictive threshold (PVE_WM_ ≥0.6)
	bias correction	yes (default)	
		no	
Reference tissue GM	RT mask	restrictive (default)	restrictive threshold (PVE_GM_ ≥0.9)
		extensive	less restrictive threshold (PVE_GM_ ≥0.6)
	bias correction	yes (default)	
		no	
Voxel	smoothing	FWHM = 10.5 mm (default)	
		FWHM = 17.5 mm	
		no	
	λ value	PVE‐weighted average (λ_pve_) (default)	$\lambda_{\text{pve}}\left(i\right)=\lambda_{\text{GM}}{\text{PVE}}_{\text{GM}}\left(i\right)+\lambda_{\text{WM}}{\text{PVE}}_{\text{WM}}\left(i\right)+\lambda_{\text{CSF}}{\text{PVE}}_{\text{CSF}}\left(i\right)$
		brain average (λ_avg_ = 0.9)	$\lambda_{\text{avg}}\left(i\right)=\lambda_{\text{GM}}\times 0.5+\lambda_{\text{WM}}\times 0.5$
		tissue‐specific (λ_tspec_)	$\lambda_{\text{tspec}}\left(i\right)=\left\{\begin{array}{c} \lambda_{\text{GM}}if\;\text{GM}\\ \lambda_{\text{WM}}if\;\text{WM}\\ \lambda_{\text{CSF}}if\;\text{CSF} \end{array}\right.$

#### 2.3.1 Generation of *M*_0_ *_t_* map

##### Long TR calibration scan (*LongTR*)

The current recommendation indicates that the *T*_1_ relaxation correction should only be carried out if TR <5 s.^4^ However, the consensus paper also recommends the use of a voxelwise approach, in which case this may not have a significant impact (as we will show). We therefore tested the impact of not correcting the *M*_0_ *_t_* images for *T*_1_ relaxation (vs. correcting, the default).

##### Control averaging (*CtrAvg*)

One of the crucial points when using the *CtrAvg* approach is the value of the TI because of its strong interaction with the *T*_1_ correction. We therefore compared using a longer (default; 2400 ms) and a shorter (800 ms) value, while correcting or not correcting for *T*_1_.

When acquisition sequences include presaturation pulses (such as Q2TIPS PASL), quantification of the presaturation efficiency, *A*, might be an important factor. For this approach, we compared A of 90% (default; the approximate average value of A estimated in *SatRec*, see below) with A of 100%.

##### Control saturation recovery (*SatRec*)

In the *SatRec* approach it is also possible to estimate *A* as part of model fitting, which should be the ideal option. Therefore, we compared estimation of *A* (default) with having a fixed *A* of 100%.

#### 2.3.2 Derivation of *M*_0_ *_a_* from *M*_0_ *_t_* map

##### Reference tissue methods (RT‐CSF, RT‐WM, RT‐GM)

Whenever using the RT method, the choice of a tissue region of interest may range between being very restrictive to being more inclusive, resulting in considerably different PVEs and potentially very different calibrations. Here, we explored the impact of using a restrictive mask (default) with less restrictive masks. In particular, a restrictive tissue mask was obtained from the respective PVE maps in ASL space, through the application of stringent thresholds (PVE_tissue_ ≥0.9). Additionally, the CSF mask was further intersected with a mask of the lateral ventricles derived from the MNI atlas.^22^ Furthermore, regarding coil sensitivity correction, we considered the bias field approach as default (see Discussion) and compared this with the option of no correction.

##### Voxelwise method (Voxel)

**Figure 1:**
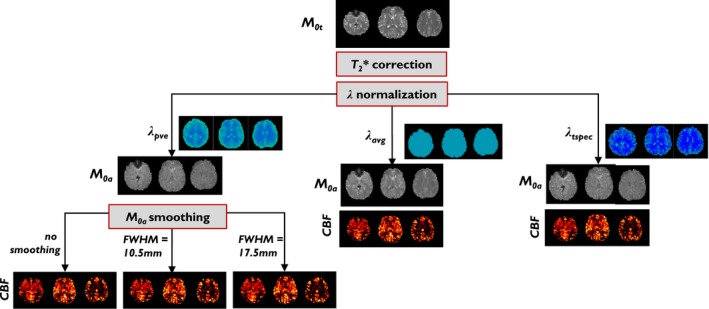
Illustrative individual example of the voxel M_0a_ derivation post‐processing options

In the Voxel method, smoothing of the *M*_0_ *_t_* map by a median filter with FWHM = 10.5 mm (3 × 3) was considered default and was compared with using a median filter with FWHM = 17.5 mm (5 × 5) as well as with the option of no smoothing. Regarding λ, the ideal PVE‐weighted λ (default) was compared with using a single brain value (0.9), the average of GM and WM λ values, and a tissue‐specific λ (Table 1). For illustration, a summary of the Voxel method post‐processing and options is displayed in Figure 1.

## 3 METHODS

The impact of the different calibration methods and respective post‐processing options was evaluated in terms of average GM CBF quantification as well as its test–retest reproducibility.

### 3.1 Data acquisition

Test–retest multiple‐PLD/TI pCASL and PASL data sets were previously acquired.^15^, ^23^ The PASL study was approved by the Hospital da Luz Ethics Committee, and all subjects gave written informed consent in accordance with the Declaration of Helsinki. The pCASL study was carried out under an agreed technical development protocol approved by the Oxford University Clinical Trials and Research Governance office, in accordance with International Electrotechnical Commission and United Kingdom Health Protection Agency guidelines.

In the PASL study, 9 healthy volunteers (22.9 ± 5.6 y, 4 males) were studied on a 3T Siemens Verio whole‐body MRI system (Erlangen, Germany) using a 12‐channel‐receive head RF coil, on 2 sessions separated by ~1 wk.^15^ In the pCASL study, 8 healthy volunteers (28.3 ± 2.5 y, 6 males) were studied on a 3T Siemens Verio whole‐body MRI system (Erlangen) using a 32‐channel receive head RF coil, on 3 occasions separated by 1 wk and 1 mo.^23^ A reference image with no labeling or background suppression, TR = 6s, and all other parameters identical to the pCASL scan, was collected for calibration. In both data sets, *T*_1_‐weighted structural images were acquired from each subject for tissue segmentation and registration purposes. Acquisition details for both data sets are summarized in Table 2.

**Table 2:** Summary of the main acquisition parameters for the PASL and pCASL data sets

	PASL (PICORE‐Q2TIPS^17^)	pCASL
Readout	2D multi‐slice GE‐EPI	2D multi‐slice GE‐EPI
Background suppression	no	yes
TR	2500 ms	4000 ms
TE	19 ms	13 ms
Number of slices	9	24
Slice time	50.0 ms	45.2 ms
Voxel size	3.5 × 3.5 × 5.0 mm^3^	3.5 × 3.5 × 5.0 mm^3^
Labeling parameters	labeling slab thickness = 100 mm	labeling duration = 1400 ms
	labeling bolus duration = 750 ms*	
	labeling slab gap = 18.8 mm	
TI/PLD values	400–2400 ms, in steps of 200 ms (11 values)	250–1500 ms, in steps of 250 ms (6 values)
Control‐label pairs	8 for each TI	8 for each PLD

* The Q2TIPS module allowed limiting the labeling to a maximum of 750 ms by adjusting TI_1_ and TI_1s_ for each TI: for TI <1000 ms, TI_1_ = TI_1s_ = TI ‐25 ms, and for TI >1000 ms, TI_1_ = 750 ms and TI_1s_ = 900 ms.

### 3.2 Data analysis

Image analysis was conducted using FSL (FSL5.0.1, http://fsl.fmrib.ox.ac.uk/fsl/fslwiki/FSL) and MATLAB (2013a, http://mathworks.com). The multiple‐PLD/TI ASL images in each data set were aligned with each other by motion correction using MCFLIRT.^24^ For each data set, at each PLD/TI, the control images and the pairwise differences between control and label images were averaged across repetitions, yielding time series of mean control images (*M_ctrl_* (PLD/TI)) and mean magnetization difference images (*ΔM_diff_* (PLD/TI)) as a function of PLD/TI, respectively. Additionally, for the PASL data set, off‐resonance effects caused by imperfect inversion slice profile in 2D multi‐slice imaging were corrected.^11^

The structural images were segmented using FAST^21^ to estimate tissue masks and corresponding partial volume estimate (PVE) maps. Both types of image were co‐registered to the ASL space of each subject and session using a linear transformation (FLIRT)^24^ (see Registration and Tissue Segmentation in the Supporting Information). For the PASL data sets, the *T*_1_ map derived from the saturation recovery approach was used as reference image, instead of the ASL data, because it provided more tissue contrast. An extended kinetic model including a tissue contribution and an intravascular arterial compartment was fitted to *ΔM_diff_* (PLD / TI), yielding maps of relative CBF and aBV, and ATT^14^, ^25^ (see Kinetic Modeling in the Supporting Information).

### 3.3 Calibration comparisons

Calibration of the data in each subject and session was carried out using the combinations of options and parameters listed in Tables 1 and 3. For the purpose of comparing calibration options, GM‐average CBF values were obtained using a GM mask derived for each subject and session using the GM mask obtained from segmentation, co‐registered to ASL space. Statistically significant differences between methods and options were tested using repeated‐measures ANOVA. When significant effects were found, post‐hoc analysis using pairwise *t* tests were carried out (*P* < 0.05). The reproducibility of the GM average CBF measurements was also assessed, by computing the inter‐ and intra‐subject coefficients of variation (CV_inter_ and CV_intra_) for the median CBF values across GM (see Coefficients of Variation in the Supporting Information).^15^, ^23^, ^26^, ^27^ Significant differences in CV across *M*_0_ *_a_* estimation methods for each *M*_0_ *_t_* map generation method were assessed using jackknife resampling, followed by 1‐way ANOVA and post‐hoc pairwise *t* tests between methods (*P* < 0.05).

**Table 3:** Parameters used in post‐processing calibration of the ASL data sets

	Parameters		
	GM	WM	CSF
*T*_1_ (s)	1.3	1.0	4.3
$T_{2}^{*}$ (ms)	60	50	400
λ	0.98	0.82	1.15

## 4 RESULTS

First, we present the quantification and reproducibility results obtained for the main calibration methods using the default post‐processing options. Second, the impact of variations in the post‐processing options is assessed.

### 4.1 Comparison between calibration methods

**Figure 2:**
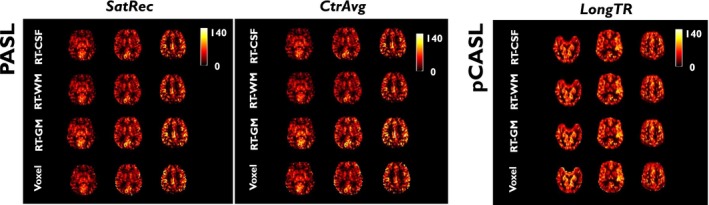
Illustrative examples of individual CBF (ml/100g/min) maps obtained using the 4 *M*_0_ *_a_* derivation methods (RT‐CSF/RT‐WM/RT‐GM/Voxel), the *SatRec* and *CtrAvg* *M*_0_ *_t_* generation methods in PASL (left), and the *LongTR* *M*_0_ *_t_* generation method in pCASL (right), with the respective default post‐processing options

Figure 2 shows illustrative examples of individual CBF maps obtained using the *SatRec* and *CtrAvg* *M*_0_ *_t_* generation methods in PASL and the *LongTR* *M*_0_ *_t_* generation method in pCASL and each of the *M*_0_ *_a_* derivation methods (RT‐CSF/RT‐WM/RT‐GM/Voxel) with the corresponding default post‐processing options. Minor differences can be observed in the Voxel compared to the RT methods, particularly in the borders of CBF maps. This can be explained by the amplification of noise that occurs on the voxelwise division by the *M*_0_ *_t_* map carried out in the Voxel method.

**Figure 3:**
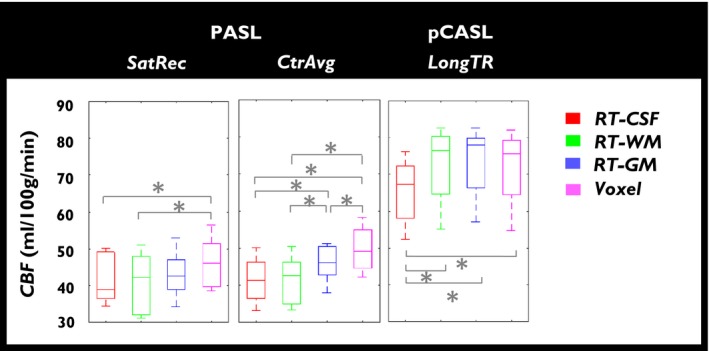
Group results for the GM average CBF values obtained using the different calibration methods for session 1 of the PASL and pCASL data sets, with the respective default options. Bottom and top edges of box plots represent the 25th and 75th percentiles, respectively. *Significant differences as assessed by pairwise *t* tests (*P* < 0.05)

The group results for the average GM CBF values, obtained using the different calibration methods with the respective default options, for the first session of each data set, are presented in Figure 3. Small CBF differences across the different *M*_0_ *_a_* derivation methods were observed although these were significantly different at times. In particular, for PASL *SatRec*, only RT‐CSF and RT‐WM methods were significantly different from the Voxel method. For PASL *CtrAvg*, CBF values were significantly different across methods except between RT‐CSF and RT‐WM methods. For pCASL *LongTR* approach, only CBF values from the RT‐CSF method were significantly different from the other *M*_0_ *_a_* derivation methods.

**Figure 4:**
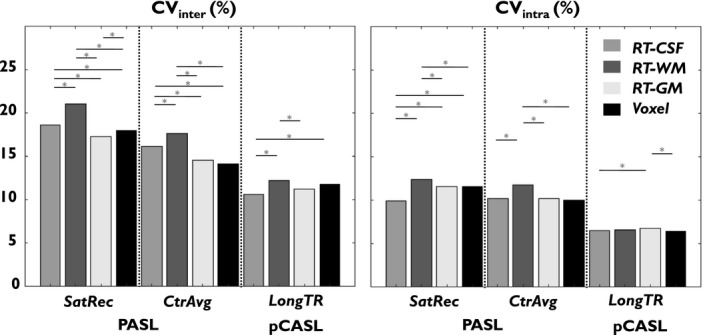
Reproducibility metrics (CV_inter_, CV_intra_) of GM average CBF values, obtained for each data set, and each of the main calibration methods, using their default post‐processing options. *Significant differences as assessed by pairwise *t* tests (*P* < 0.05)

Finally, the reproducibility metrics of the average GM CBF values for the main calibration methods, using the default post‐processing options, are presented in Figure 4. All methods were found to exhibit good reproducibility, with pCASL being superior to PASL, and the RT‐WM method generally yielding the worst values. A significant main effect of *M*_0_ *_a_* derivation method was found for each *M*_0_ *_t_* map generation method. Subsequent post‐hoc analysis yielded significant differences between several *M*_0_ *_a_* derivation methods. The Voxel and RT‐CSF methods yielded the lowest CV_intra_ values, whereas the Voxel and RT‐GM methods yielded the lowest CV_inter_ values, with significant differences in several cases. The RT‐WM method systematically carried out worst for both CV_intra_ and CV_inter_, often with significant differences.

### 4.2 Impact of calibration post‐processing options

**Figure 5:**
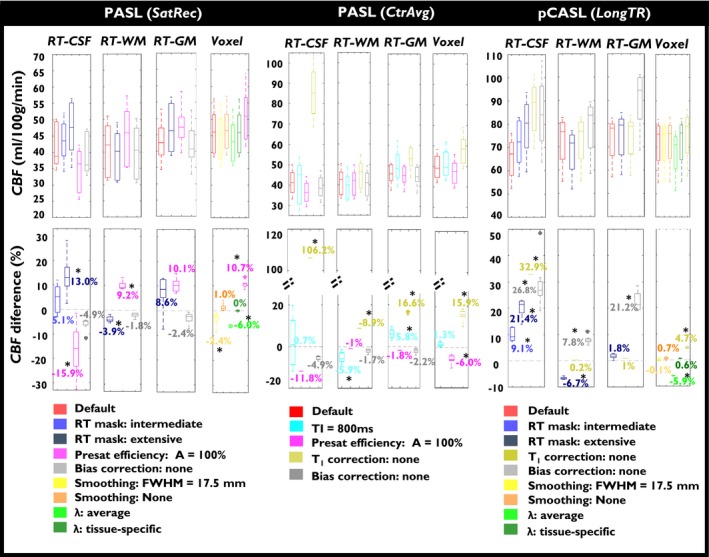
Group results for the GM average CBF values (top) and differences relative to default options (bottom), obtained when varying the calibration post‐processing options, for each of the main calibration methods tested, in session 1 of PASL‐*SatRec* (left), PASL‐*CtrAvg* (middle), and pCASL‐*LongTR* (right). Box plots represent the 25th and 75th percentiles, respectively. *Significant differences as assessed by pairwise t tests (*P* < 0.05)

The group results for the GM average CBF values obtained when varying the calibration post‐processing options are presented in Figure 5, for each of the main calibration methods tested on session 1 of each data set. For each method, there was a significant main effect of the post‐processing option; subsequent pairwise comparisons revealed significant differences between some options, as indicated in the plots.

When applying the *SatRec* method for *M*_0_ *_t_* generation, the RT‐CSF approach yielded the highest CBF differences across methods, with the extensive mask producing significantly greater CBF values relative to the default option that uses a much more restrictive mask. WM masking options resulted in a significant CBF decrease, but only of ~5% relative to the default value. Fixing *A* = 100% instead of allowing its estimation (*A* ~90%) yielded significant CBF differences relative to default option for all methods except for RT‐GM. Not correcting for field inhomogeneities using bias correction had small and non‐significant impact on CBF quantification, although the impact was higher when using the RT‐CSF approach compared with the other RT approaches. For the Voxel approach, an increase in kernel size for spatial smoothing led to significant but small effects on CBF. Not applying any smoothing at all yielded non‐significant differences. Finally, different options for the value of λ also resulted in significant but small CBF differences when using an average λ, whereas non‐significant differences were observed when using a tissue‐specific λ.

In contrast with the *SatRec* approach, the *CtrAvg* approach revealed great sensitivity to some post‐processing options, particularly the *T*_1_ correction, and more so when using the RT‐CSF method. Significant increases in CBF as high as ~100% were found when using the RT‐CSF method if no *T*_1_ correction was carried out. When using TI = 800 ms, significant CBF differences were found for RT‐WM and RT‐GM methods compared to the default option.

When applying the pCASL *LongTR* method for *M*_0_ *_t_* generation, differences in CBF values were obtained with the different options, particularly when using the RT‐CSF method. When not performing *T*_1_ correction, the RT‐CSF method yielded a significantly greater difference compared with the default options, with the Voxel method yielding a significant but much smaller difference. Consistently with the PASL *SatRec* results, less restrictive masks led to important and significant CBF differences, except for the RT‐GM method, particularly in the case of the extensive CSF mask. Also, similarly with PASL *SatRec* but to a higher extent, bias correction had greater and always significant impact on all RT methods, particularly on RT‐CSF and RT‐GM. When using the Voxel method, the different spatial smoothing options yielded non‐significant differences relative to the default option. Non‐significant differences were also observed when using a tissue‐specific λ but, in contrast, average λ yielded a statistically significant difference.

## 5 DISCUSSION

We systematically compared the main ASL calibration methods as well as the associated post‐processing options, in terms of CBF values and corresponding test–retest reproducibility.

### 5.1 Comparison between calibration methods

When comparing the different calibration methods using the default post‐processing options, minimal differences were observed in CBF within each data set (Figure 2). When focusing on the GM average CBF values (Figure 3), relatively small differences were found despite being significant in some cases.

Overall, the RT‐CSF method yielded the lowest CBF values across *M*_0_ *_t_* estimation methods. Although, for the PASL *SatRec* approach, only RT‐CSF and RT‐WM methods were significantly different from the Voxel method, for the pCASL *LongTR* approach, significantly lower CBF values were obtained when using the CSF as a reference tissue, compared with other strategies for deriving *M*_0_ *_a_* from *M*_0_ *_t_*. This finding may be explained by the fact that our pCASL data set, obtained using a 32‐channel head coil, suffered from B_1_ field inhomogeneities that were not fully accounted for by bias field correction. In fact, this interpretation is corroborated by the observation that, in the case of pCASL, when using less restrictive CSF masks (including the whole lateral ventricles rather than just a few central voxels where coil sensitivity is much lower), the CBF values increased considerably and became comparable to the ones obtained using the Voxel approach.

It should be noted that the bias field correction approach used here is not the ideal method for accounting for the coil sensitivity profile. Nevertheless, this strategy serves the purpose of evaluating the relative differences between calibration methods and post‐processing options. In fact, it clearly emphasizes the impact of sensitivity correction on CBF, particularly when using the RT‐CSF approach. Furthermore, the CBF values obtained with RT methods, when corrected for the bias field, become closer to the ones obtained with the Voxel method, which intrinsically corrects for this issue. Nevertheless, further work is necessary to fully estimate the impact of this strategy on calibration methods. Methods for the estimation of the coil sensitivity profile have been proposed,^28^, ^29^ but these require the acquisition of additional data using imaging sequences that are not widely available. Alternatively, a proton density image acquired with a body coil can also be used to obtained an approximate sensitivity map.^30^ However, this method is limited by the fact that it only corrects for non‐uniformity in the receive field, not the transmit field, which is also not spatially uniform at field strengths of 3T or higher, and this correction may not account for other factors (e.g., prescan transmit settings).

It is possible to circumvent RF field inhomogeneity issues altogether by using the voxelwise method. In this case, *M*_0_ *_a_* is computed at each voxel based on the measured *M*_0_ *_t_* image and it is therefore affected by the same RF coil sensitivity as the ASL measurements themselves. Normalization of the estimated CBF map by the *M*_0_ *_a_* map therefore intrinsically corrects for coil sensitivity variations. This well‐known effect has been explicitly reported,^20^ with observed signal loss in anterior regions on both proton‐density and perfusion‐weighted ASL images being successfully restored in the final perfusion maps by calibration using a Voxel approach. Nevertheless, the voxelwise division intrinsic to this approach might lead to increased variability in CBF quantification because of noise amplification and also to edge and/or partial volume effects.^31^

### 5.2 Impact of calibration post‐processing options

We observed that some of the processing options had great impact on the final results whereas others were relatively unimportant. Interestingly, very few studies have investigated the impact of different calibration strategies, and critically, they are not in agreement. One study compared the 3 *M*_0_ *_a_* derivation schemes that we also tested here (RT‐CSF, RT‐WM, and Voxel) using data collected with 3 single‐TI (1400 ms) PASL sequences (including PICORE‐Q2TIPS) and found ~35% higher CBF values for the RT‐CSF relative to the Voxel method.^18^ Another study also observed a discrepancy in CBF quantification when using different calibration methods based on single‐PLD pCASL data, with RT‐WM yielding the lowest CBF values and Voxel the highest (~20% difference).^20^ A third study compared different calibration methods on CASL and pCASL data and found only minor differences. However, they reported slightly lower CBF values using RT‐CSF compared with RT‐WM possibly because of not correcting for the uneven sensitivity profile of the head coil.^19^

Our results highlight that one of the processing options with greatest impact was the correction of the *M*_0_ *_t_* map for incomplete *T*_1_ relaxation in each tissue based on the TR of the respective image acquisition, particularly when using the RT‐CSF method. In the current guidelines, this correction is only recommended when TR <5 s.^4^ However, this recommendation is coupled with the use of the Voxel method for extrapolating *M*_0_ *_a_*, in which case we do not observe large effects of *T*_1_ correction. In fact, the impact of *T*_1_ correction is much greater if the RT‐CSF derivation method is used, because CSF has a much longer *T*_1_ than GM or WM. In this case, a TR longer than 16 s would be required for an almost full recovery of the longitudinal magnetization. Therefore, we believe that this correction should be applied even if TR is longer than 5 s, when the RT‐CSF calibration method is used. A related option with relatively high impact is the value of PLD/TI used to extract *M*_0_ *_t_*. In the case of the *CtrAvg* approach in PASL, using a lower TI leads to greater sensitivity in CBF quantification. For PASL, the presaturation efficiency value was also important, because this is sequence‐specific and therefore should be chosen accordingly. The impact of the bias correction approach is highly dependent, not only on the *M*_0_ *_t_* derivation method, but also on the type of ASL data set and the RF coil sensitivity of the acquisition. In contrast with the 12‐channel RF coil used for the PASL data set, the 32‐channel RF coil used for the pCASL data set displayed a relatively heterogeneous sensitivity profile, leading to significant impact of bias correction, particularly when using RT‐CSF and RT‐GM methods.

**Figure 6:**
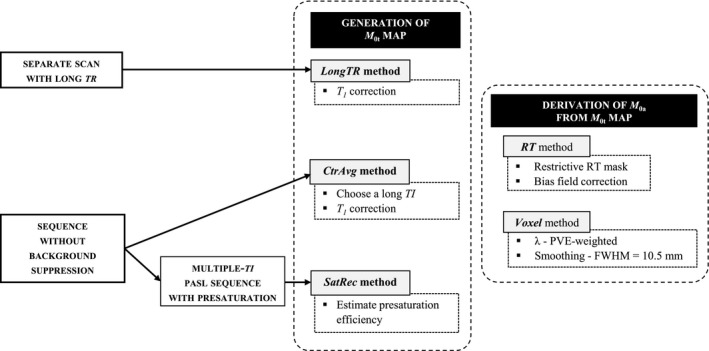
Summary of the main calibration methods, according to the data acquisition procedure (left) and with indication of the corresponding recommended options, for the generation of the *M*_0_ *_t_* map (middle) and the derivation of *M*_0_ *_a_* from the *M*_0_ *_t_* map (right)

Overall, our results indicate that when acquiring multiple‐TI PASL data, the *SatRec* approach is preferable to the *CtrAvg* approach because the latter strongly depends on the choice of PLD/TI value and on *T*_1_ correction. In terms of *M*_0_ *_a_* derivation, the RT‐CSF approach was the most sensitive to variations in the processing options, making this approach more prone to CBF discrepancies. In addition, variability in the CBF differences across subjects was also greater than with other *M*_0_ *_a_* derivation methods. The RT‐CSF method is highly dependent, not only on *T*_1_ correction as discussed before, but also on the tissue mask and whether or not bias correction is carried out. The RT‐WM and RT‐GM methods are also dependent on these parameters but to a lesser extent. Notwithstanding, the RT‐WM approach might not be feasible whenever WM lesions are present, such as in multiple sclerosis or small vessel disease. On the other hand, the RT‐GM approach is highly influenced by PVE, rendering the use of restrictive GM masks mandatory. Nevertheless, approaches for PVE correction most often target only the relative CBF images and not *M*_0_ measures.^31^, ^32^ Therefore, we believe that this correction should not affect our results concerning the comparison of calibration methods. Similarly to the RT‐WM and RT‐GM methods, the Voxel method yielded relatively small CBF differences across different processing options. In fact, some options had negligible impact on CBF quantification, namely the degree of smoothing. As expected, the use of an averaged, fixed λ underestimates GM CBF. A summary scheme of recommended calibration procedures, as well as the corresponding options that should be applied and/or reported, is depicted in Figure 6.

### 5.3 CBF values, spatial distributions and reproducibility

The maps obtained in our study exhibit spatial distributions that are consistent with the expected variations across tissues and brain regions.^5^, ^15^, ^33^ All average GM CBF values found in our study were within the wide range of values reported in the literature. However, the PASL data set systematically produced lower CBF values than the pCASL data set. Surprisingly, in a study using 5 different commonly used ASL sequences, the opposite pattern was seen between single‐PLD pCASL and multiple‐TI PASL using the RT‐CSF method (25/40 mL/100 g/min for pCASL/PASL).^6^ In another study, similar values were found for both PASL and pCASL single‐PLD/TI sequences (62/60 mL/100 g/min for PASL/pCASL), using different calibrations strategies (extra calibration scan for PASL/control averaging for pCASL).^34^ The differences between pCASL and PASL observed here are of unknown cause and are likely explained partially by specific implementation issues.

The inter‐ and intra‐subject coefficients of variation obtained in our study for GM average CBF are all within intervals of good reproducibility. For PASL, CV_intra_ ~10–12% is in agreement with the literature. A multiple‐TI QUASAR ASL study using a model‐free approach reported within‐week CV_intra_ ~10%,^35^ whereas a study from our group achieved within‐week CV_intra_ values in the range of 10–21%.^15^ A single‐TI PASL study, using a sequence similar to the 1 used in our study, found within‐week CV_intra_ ~6%.^5^ Regarding pCASL, our results of ~6–7% are also in accordance with the literature. A single‐PLD pCASL study using 2 different PLD values yielded CV_intra_ ~5–10%, which increased with PLD.^36^ Consistently, a multiple‐PLD pCASL study obtained CV_intra_ ~5% for average week‐repeat GM CBF value.^23^

### 5.4 Limitations and future work

Although our approach aimed to systematically and comprehensively test all relevant processing options in the calibration methods used, options regarding relatively less important parameters could also be tested, in particular the tissue‐specific $T_{2}^{*}$ used to correct for $T_{2}^{*}$ decay when deriving the *M*_0_ *_t_* maps. Another option that could be tested is the use of subject‐specific or voxelwise *T*_1_ values, which could be estimated directly when using the *SatRec* approach in PASL or acquired separately. Importantly, some of the calibration methods may not be appropriate to use in certain clinical populations. For example, in patients with WM abnormalities, such as in multiple sclerosis or small vessel disease, the RT‐CSF or RT‐GM methods may be preferable to the RT‐WM.

Furthermore, we acknowledge that some options could not be tested because of the lack of data availability. In particular, the *LongTR* approach should be also compared using PASL, to determine the best method for *M*_0_ *_t_* generation. Additionally, a more precise approach for correction of the coil sensitivity profile should be carried out. Furthermore, we also acknowledge that the EPI images suffer from susceptibility‐induced geometric distortions that might affect image co‐registration. This can be circumvented by acquiring a B_0_ field map and using this to correct such distortions. Finally, a prospective study assessing the impact of calibration strategies by acquiring both PASL and pCASL on the same subjects should be conducted to further validate our results.

## 6 CONCLUSION

In conclusion, we found that considerable discrepancies in CBF values can be obtained when using poor choice simplifications in the post‐processing calibration of pCASL and PASL data. Nevertheless, strategies based on reference tissue or voxelwise methods both perform well when carefully implemented in terms of CBF quantification and reproducibility. In general, the greatest sensitivity was found for correction for incomplete *T*_1_ relaxation when using proton density reference images for calibration. Correction for RF field inhomogeneities also had great impact, as did the value of presaturation efficiency, whereas the values of brain–blood water partition coefficient had moderate impact. In contrast, the degree of spatial smoothing applied to the calibration images or the mask used for the reference tissue had minor effects. The voxelwise approach, as proposed in the ASL white paper, has the advantage of simplicity, while the reference tissue methods are more vulnerable to sensitivity and relaxation correction errors, in particular when using CSF. Nevertheless, our results emphasize the need for consistent post‐processing options across studies as well as the need for a complete description of the various post‐processing calibration options so that absolute CBF quantification is effectively achieved. Failure to take the impact of these fine calibration options into account would seriously compromise the use and applicability of ASL perfusion imaging.

## Supporting information


**FIGURE S1** Illustrative examples of MPRAGE images registered to ASL space (bottom) and respective CSF (blue) and GM (yellow) masks retrieved from MPRAGE image segmentation (top), for the PASL and pCASL datasets

## Data Availability

The underlying data associated with figures in this study are available from the Oxford University Research Archive (ORA‐Data) (DOI: https://doi.org/10.5287/bodleian:xQx8Vnv5e). For enquiries regarding the imaging data contact: ARM for the pCASL data (andrew.segerdahl@ndcn.ox.ac.uk) or PF for the PASL data (patricia.figueiredo@tecnico.ulisboa.pt).
