# Supplementary material for: Calibration of arterial spin labeling data—potential pitfalls in post‐processing
Source: Magn Reson Med. 2019 Oct 12;83(4):1222–34. doi: 10.1002/mrm.28000 (PMC6972489; doi:10.1002/mrm.28000)
Supplement: Supplementary file 1 — FIGURE S1 Illustrative examples of MPRAGE images registered to ASL space (bottom) and respective CSF (blue) and GM (yellow) masks retrieved from MPRAGE image segmentation (top), for the PASL and pCASL datasets [file MRM-83-1222-s001.docx]

**Supporting Information**

***Kinetic Modelling***

The tissue component (${\Delta M}_{tiss}$) was described by the general kinetic model^1^:

| PASL | ${\Delta M}_{tiss}(t)={\alpha M}_{0a}\frac{2f}{k}\left\{ \begin{matrix} 0 \\ e^{-t/T_{1a}}\left( e^{k\left( t-ATT \right)}-1 \right) \\ e^{-t/T_{1a}}\left( e^{k\left( t-ATT \right)}-e^{k\left( t-BAT-\tau\right)} \right) \end{matrix}\begin{matrix} if \\ if \\ if \end{matrix} \right.\begin{matrix} t< ATT \\ ATT\leq t<ATT+\tau\\ t \geq ATT+\tau\end{matrix}$ | (Eq. 1) |
| --- | --- | --- |
| pCASL | ${\Delta M}_{tiss}\left( t \right)={\alpha M}_{0a}2f T_{1}^{'} \left\{ \begin{matrix} 0 \\ e^{-ATT/T_{1a}}\left( {1-e}^{(t-ATT)/T_{1}^{'}} \right) \\ e^{-ATT/T_{1a}}\left( e^{-(t-\tau-ATT)/T_{1}^{'}}-e^{-(t-ATT)/T_{1}^{'}} \right) \end{matrix}\begin{matrix} if \\ if \\ if \end{matrix} \right.\begin{matrix} t< ATT \\ ATT\leq t<ATT+\tau\\ t \geq ATT+\tau\end{matrix}$ | (Eq. 2) |

with $k=\frac{1}{T_{1b}}-\frac{1}{T_{1}^{'}}$, $\frac{1}{T_{1}^{'}}=\frac{1}{T_{1}}+\frac{f}{\lambda}$, where ${\Delta M}_{tiss}$ is the control-label magnetization difference measured from the tissue compartment; $M_{0a}$ is the equilibrium magnetization of the arterial blood; $\tau$ is the bolus duration; $T_{1a}$ is the longitudinal relaxation time of arterial blood; $T_{1,t}$ is the longitudinal relaxation time of brain tissue;$\lambda$ is the blood-brain water partition coefficient, $\alpha$ is the labeling (inversion) efficiency, and $t$ corresponds to *TI* for PASL and $\tau+PLD$ for pCASL. The intravascular arterial component (${\Delta M}_{art}$) was added as^2,3^:

| PASL | ${\Delta M}_{art}\left( t \right)=\alpha M_{0a}2aBV\left\{ \begin{matrix} 0 \\ e^{-t/T_{1a}} \\ 0 \end{matrix}\begin{matrix} if \\ if \\ if \end{matrix} \right.\begin{matrix} t< ATTa \\ ATTa\leq t<ATTa+\tau_{a} \\ t \geq ATTa+\tau_{a} \end{matrix}$ | (Eq. 3) |  |
| --- | --- | --- | --- |
| pCASL | ${\Delta M}_{art}\left( t \right)=\alpha M_{0a}2aBV\left\{ \begin{matrix} 0 \\ e^{-ATTa/T_{1a}} \\ 0 \end{matrix}\begin{matrix} if \\ if \\ if \end{matrix} \right.\begin{matrix} t< ATTa \\ ATTa\leq t<AATa+\tau_{a} \\ t \geq ATTa+\tau_{a} \end{matrix}$ | (Eq. 4) | |

where *ATTa* is the intravascular arterial transit time; and $\tau_{a}$ is the intravascular bolus duration. Finally, the total magnetization difference ${\Delta M}_{total}\left( t \right)= {\Delta M}_{tiss}\left( t \right)+{\Delta M}_{art}(t)$ was fitted to the ${\Delta M}_{diff}(t)$ time series measured in each voxel using BASIL^2,3^.

***Coefficients of Variation***

CV_inter_ was computed for each session as:

|  | $CV_{\mathrm{inter}}= \frac{\mathrm{SD}}{\mu}\times100 [\%]$ | (Eq. 5) |
| --- | --- | --- |

where μ and SD are the mean and standard deviation (SD) of the parameter across subjects ^4^. The final CV_inter_ was computed as the mean of the CV_inter_ of the two sessions. CV_intra_ was computed as:

|  | $CV_{\mathrm{intra}}=\frac{SD_{\mathrm{ws}}}{\mu}\times100 [\%]$ | (Eq. 6) |
| --- | --- | --- |

where $\mu$ is the mean value of the parameter across subjects and sessions ^4^. SD_ws_ is the standard deviation of repeated measurements within-subject, computed as:

|  | $SD_{\mathrm{ws}}=\sqrt{\left( \frac{\sum_{i=1}^{k} \left( a_{{R_{i}S}_{1}-}a_{{R_{i}S}_{2}} \right)^{2}}{2 \times k} \right)}$ | (Eq. 7) |
| --- | --- | --- |

where k is the number of subjects and $a_{{R_{i}S}_{1}}$ and $a_{{R_{i}S}_{2}}$ are the measurements of subject i on session 1 and 2, respectively.

***Registration and Tissue Segmentation***


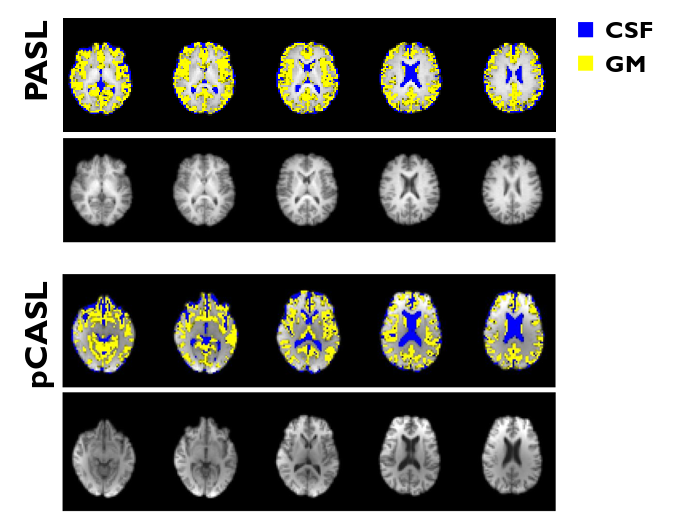
 **Supporting Information Figure S1**. Illustrative examples of MPRAGE images registered to ASL space (bottom) and respective CSF (blue) and GM (yellow) masks retrieved from MPRAGE image segmentation (top), for the PASL and pCASL data sets.

**References:**

1. Buxton RB, Frank LR, Wong EC, et al. A general kinetic model for quantitative perfusion imaging with arterial spin labeling. *Magn Reson Med* 1998; 40: 383–96.

2. Chappell MA, Groves AR, Whitcher B, et al. Variational Bayesian Inference for a Nonlinear Forward Model. *IEEE Trans Signal Process* 2009; 57: 223–236.

3. Chappell MA, MacIntosh BJ, Donahue MJ, et al. Separation of macrovascular signal in multi-inversion time arterial spin labelling MRI. *Magn Reson Med* 2010; 63: 1357–65.

4. Bland JM, Altman DG. Statistics Notes Measurement error. 1996; 313: 1996.
